# Exfoliative Cytology and Genetic Analysis for a Non-Invasive Approach to the Diagnosis of White Sponge Nevus: Case Series

**DOI:** 10.3390/bioengineering10020154

**Published:** 2023-01-23

**Authors:** Carlo Lajolo, Concetta Cafiero, Egidio Stigliano, Francesca Romana Grippaudo, Pietro Chiurazzi, Cristina Grippaudo

**Affiliations:** 1 Head and Neck Department, Università Cattolica del Sacro Cuore, Fondazione Policlinico Universitario A. Gemelli IRCCS, Largo A. Gemelli 8, 00168 Rome, Italy; 2 Area of Molecular Pathology, Anatomic Pathology Unit, Fabrizio Spaziani Hospital, Via Armando Fabi 2, 03100 Frosinone, Italy; 3 Area of Pathology, Department of Woman and Child Health and Public Health, Università Cattolica del Sacro Cuore, Fondazione Policlinico Universitario A. Gemelli IRCCS, Largo A. Gemelli 8, 00168 Roma, Italy; 4 Plastic Surgery Unit, Faculty of Medicine and Psychology, Sapienza University of Rome, 00185 Rome, Italy; 5 Medical Genetics Unit, Institute of Genomic Medicine, Università Cattolica del Sacro Cuore, Fondazione Policlinico Universitario A. Gemelli IRCCS, Largo A. Gemelli 8, 00168 Roma, Italy

**Keywords:** White Sponge Nevus (WSN), incisional biopsy, liquid-based cytology, Cell Block, *KRT4*, *KRT13*

## Abstract

Background: White Sponge Nevus (WSN) is a rare benign disorder associated with mutations in genes coding for cytokeratin 4 (KRT4) and 13 (KRT13) characterized by dyskeratotic hyperplasia of mucous membranes. This study was aimed at examining different approaches (cytology, pathology and genetic analysis) to WSN diagnosis. Methods: A series of four patients with asymptomatic white diffuse oral lesions were evaluated and, before performing an incisional biopsy for pathology, an oral brush Thin Prep was collected for exfoliative liquid-based cytology (LBC). DNA for genetic analysis was also obtained from patients and both their parents, using buccal swabs. Results: Pathology and cytology showed similar results, leading to the same diagnosis of hyperkeratotic epithelium with acanthosis and spongiosis, without atypia, demonstrating the efficiency of LBC for the differential diagnosis. Sequencing analysis revealed at least 6 rare variants in the *KRT4* and *KRT13* genes in each patient, contributed in part by both unaffected parents. Conclusions: Thin Prep for oral exfoliative cytology and genetic analysis are sufficient for an accurate diagnosis of WSN. The combination of cytological and genetic analyses could substitute the histologic exam, providing a non-invasive alternative for incisional biopsy.

## 1. Introduction

Oral cavity can be affected by many different benign, premalignant and malignant diseases that clinically appear as white patches. White Sponge Nevus (WSN) is a rare mucosal leukokeratosis, occurring in less than 1 in 200,000 births, that can be transmitted in a dominant fashion in association with pathogenic variants of the *KRT4* and *KRT13* genes encoding keratin 4 and 13 [1,2,3]. WSN predominantly affects the oral mucosa (although vagina, rectum, and nasal cavity may be similarly involved) and is characterized by dyskeratotic hyperplasia of mucous membranes, usually presenting bilateral, symmetric, asymptomatic, deeply fissured oral white patches; oral lesions can arise in different parts of the oral mucosa, with buccal mucosa being more frequently affected [4,5]. Lesions are thick and spongy, typically appear before puberty, and tend to proliferate diffusely substituting the normal epithelium [6,7]. For this reason, even if it is a benign condition, patients and their parents, in case of young subjects, are worried.

WSN is listed in the OMIM (Online Mendelian Inheritance in Man) catalogue as a rare autosomal dominant benign disorder (see OMIM entries #193900 and #615785) with incomplete penetrance and variable expression, thus familial cases are rare [8,9,10]. It is caused by germline variants of the keratin genes *KRT4* (OMIM *123940) or *KRT13* (OMIM *148065) located on chromosomes 12q13 and 17q21-q22, respectively [8]. These genes encode keratin intermediate filament proteins KRT4 and KRT13, that are specific for mucosa and have been shown to be implicated in WSN development and progression [11,12,13]. 

The differential diagnosis is necessary when an hereditary epithelial disorder is supposed, in fact many other genodermatoses (Table 1), such as Hereditary Benign Intraepithelial Dyskeratosis (OMIM %127600) and Follicular Keratosis (Darier-White disease, OMIM #124200), can affect the oral cavity and should be excluded [1,2,14].

Patients discovering the presence of WSN while they are young adults or older pose difficult differential diagnosis, since some potentially malignant disorders can present as white patches [1,15,16]: conditions such as leukoplakia (especially its proliferative verrucous form) and oral lichen planus (in its plaque clinical form) must be excluded; furthermore, other benign white lesion must be excluded as leukoedema, lichenoid drug reaction, lupus erythematosus, cheek chewing, muscles and candidiasis [17,18,19]. In all the above-mentioned diseases, it is extremely important to reach the correct diagnosis and eventually start a suitable therapy. Even if a biopsy is usually indicated to distinguish WNS and there are few contraindications, Morris et al. [13] reported a case with WSN, where the diagnosis was confirmed by ultrastructural cytology, thus the possibility to reach a correct diagnosis with a non- invasive technique should be explored especially when WSN must be excluded in children. 

In the last decade, cytopathology alone and/or in combination with additional analyses (i.e., immune-cytopathology, metabolic assays, genetic sequencing), have been widely used in all fields of medicine. Recently some studies reported its potential use in the diagnosis of oral diseases, and some interesting results have been reached for the early diagnosis of oral cancer and follow-up of potentially malignant disorders [20,21]. Over the past ten years, cytology and specifically cytoinclusion (Cell-Block) has proven to be a highly reliable diagnostic technique for the diagnosis of oral premalignant and malignant lesions, such as oral squamous cell carcinomas. Furthermore, its non-invasive nature and the possibility to perform ultrastructural and molecular assays make this technique very useful especially in children or in severely compromised patients [22,23].

Since WSN is generally diagnosed in adolescence, from a clinical point of view it should be differentiated mainly from thermal injuries, oral pseudomembranous candidiasis, trauma (factitia) and more rarely from oral lichen planus and other lichenoid lesion. Compared to thermal injuries, WSN has stable lesions over time, diffused in many anatomical oral district [24]. Unlike oral lichen planus and other lichenoid lesions, WSN does not present the typical Wickham striae and cannot be removed with a gauze or a spatula as candidiasis. 

The aim of this study is to report 4 new cases of WSN, comparing the results from non-invasive diagnostic techniques (oral brush Thin Prep for exfoliative cytology and sequencing of *KRT4* and *KRT13* genes) with the results of mucosal membrane biopsy, in order to evaluate the usefulness of non-invasive diagnostic tools. Eventually we propose that the combination of the two non-invasive methods should substitute the use of biopsy in the diagnosis of WSN.

## 2. Materials and Methods

Four male patients, aged 14, 19, 22 and 37 respectively, were referred to the Dental Clinic of Fondazione Policlinico Gemelli (FPG), Catholic University of Rome, for the diagnosis of white asymptomatic diffuse oral lesions. They were all selected for this study, because the aspect of the lesions led to the suspicion of WSN; given its rarity, the number of patients was considered sufficient for this observational study. The study was approved by the Ethic Committee of the Università Cattolica del Sacro Cuore (#565). A written consent, including information about the diagnostic procedures i.e., oral biopsy, exfoliative cytology and genetic testing, was given to all patients, and to the parents of the minor. 

### 2.1. Patient Information and Clinical Findings

The first patient was sent to our observation from a dermatologist, who had suspected WSN, in order to evaluate the possibility of orthodontic therapy. The second patient was referred from his dentist; the third and the fourth came from the dermatological Clinic of FPG. None of the patients were smokers and their medical history did not reveal any systemic diseases nor pharmacological treatments. 

Extra-oral examination did not reveal any contributory feature and the intraoral examination revealed in all cases white diffuse spongy lesions mainly located on the buccal mucosa, affecting also the tongue and floor of the mouth. None of the family members of these patients presented any white lesion of the mouth. In all cases white spongy smooth patches were present bilaterally on the cheek mucosa (from retro-commissural area to the trigone area) and in the ventral surface of the tongue. The dorsum and the border of the tongue were only slightly more spongy than normal for case 1 and 2 and 4, while this was the case only for the ventral surface of the tongue in case 3. The soft or hard palate were not involved in any case. In all cases the Nikolsky sign was negative, but a slight white keratin membrane could be removed from the most outer part of the mucosal membrane (especially on the buccal mucosa). 

### 2.2. Timeline

After clinical examination, a smear was gently collected from the lesion using a cytobrush for oral exfoliative cytology procedures (ThinPrep kit, Hologic Inc., Mississagua, ON, Canada), without damaging the lesion. The samples for cytological tests from each patient were immediately deposited into 20 mL of PreservCyt^®^ Solution (Hologic Inc., Mississagua, ON, Canada). The sample vials were then capped, labeled, and sent to the laboratory of cytopathology of FPG. The samples were analyzed the same day of the laboratory delivery, however the PreservCyt^®^ Solution allows sample storage for 30 days. Three non-invasive laboratory procedures were performed on each sample in order to verify the diagnosis of WSN: Papanicolau staining for cytomorphologic determination (ThinPrep), immune-cytopathology and Cell Block (CB) preparation [16,25]. 

A second sample was taken by an oral brush (Cytobrush^®^ Plus GT, Cooper surgical, Trumbull, CT, USA) for genetic analysis; this sample was stored dry and sent to the Genetic Laboratory of FPG for genetic analysis.

The diagnostic pathway ended with an incisional biopsy of the white lesions for the pathology diagnosis. After performing chlorhexidine 0.2% disinfection of the oral mucosa (Paroex, GUM, Sunstar, Etoy, Switzerland) and local anesthesia with 2% mepivacaine with 1:100,000 epinephrine (Optocain, Molteni Dental, Milan, Italy), a 6 mm punch biopsy was taken (kai Europe GmbH, Solingen, Germany). A 3/0 silk (Ethilon, Ethicon, Johnson & Johnson Medical Spa, Pomezia, Italy) was used to suture the wound; in all patients stitches were removed after one week, and healing was uneventful. All specimens were formalin fixed and sent to the pathology laboratory of FPG, with a clinical diagnosis of WSN.

### 2.3. Diagnostic Assessment

**Figure 1:**
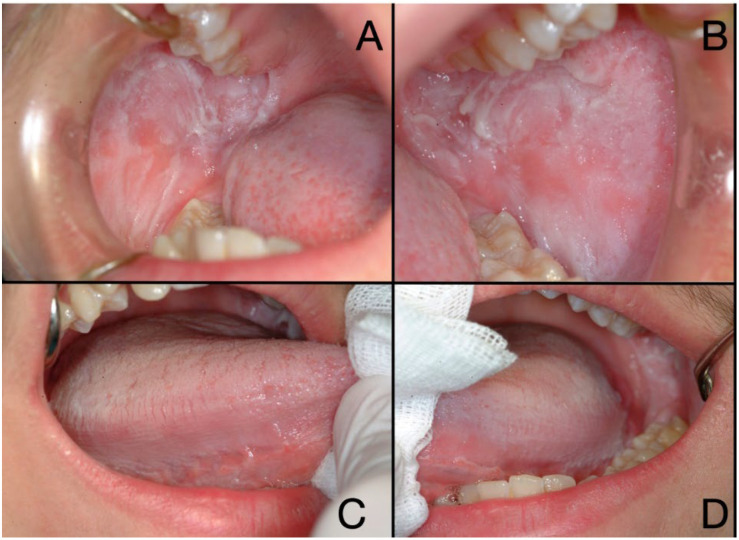
Clinical appearance of the same patient (right cheek (**A)**, left cheek (**B**), right tongue (**C**), left tongue (**D**) with White Sponge Nevus: the mucosa appears bilaterally whitish, thickened and spongy texture.

Medical history, young age (except one patient—37 years old) and clinical appearance of patients oral mucosa were highly suggestive of WSN (Figure 1), nevertheless we had the opportunity to perform non-invasive diagnostic pathology procedure (cytopathology, immune-cytopathology and cell block) together with genetic tests and compare them with the standard diagnostic procedure based on bioptic tissue examination.

### 2.4. Laboratory Procedures

Part of the buccal smear sample was prepared using Hologic Thin Prep 5000 (Hologic Inc., Mississagua, ON, Canada) and stained with the trichrome of Papanicolau (Hologic Inc., Mississagua, ON, Canada) for cytopathologic analysis. From the same smear sample, slides were prepared for immunostaining with antibodies against keratins KRT4 and KRT13 (Santa Cruz Biotechnology, Dallas, TX, USA), to evaluate their relative expression. The remaining part of the smear samples were prepared using the Celliant Automated Cell Block system (Hologic Inc., Mississagua, ON, Canada). This kind of preparation allows observing a thick layer of cells. The Cell Block were stained with hematoxylin and eosin (H&E) and immunostaining with antibodies against KRT4 and KRT13. The incisional biopsy specimens obtained from the buccal mucosa of all patients were routinely processed as specimens for H&E staining and immunostaining with antibodies against KRT4 and KRT13.

### 2.5. Genetic Analysis

DNA extraction and purification was carried out using an automatic MagCore Nucleic Acid Extractor. DNA concentration and quality were measured by absorbance at 260 nm and by the ratios of A260 nm/A280 nm. Polymerase chain reaction (PCR) primers were designed using Genamics Expression DNA Sequence Analysis Software (Genamics, Hamilton, New Zealand) and the in Silico-PCR tool provided by the UCSC Genome Browser (http://rohsdb.cmb.usc.edu/GBshape/cgi-bin/hgPcr, accessed on 29 September 2021). Primer sets were designed to include all coding exons (9 for *KRT4* and 8 for *KRT13*) and splice junctions, including a minimum of 50 nucleotides of intron sequence (Table 2). PCR amplification of all exons was performed using PCR Master Mix (Promega Corporation, Madison, WI, USA). 

PCR cycles consisted of denaturation at 95 °C for 2 min, 38 cycles of 95 °C for 45 s, 55 °C for 45 s, 72 °C for 45 s, and a final elongation at 72 °C for 5 min. PCR products were purified using ExoStar1 Step (Euroclone S.p.A. Società a Socio Unico Via Figino, 20/2220016 Pero (MI)), directly sequenced on both strands using BigDyeTerminator V3.1 (ThermoFisher Scientific, Waltham, MA, USA) and subsequently resolved on ABI3500 Genetic Analyzer (Applied Biosystems, Foster City, CA, USA). NM_002272.4 was used as reference sequence of the *KRT4* gene and NM_153490.3 was used as reference sequence of the *KRT13* gene. Identified variants were confirmed with a new PCR and sequencing reaction. 

### 2.6. In Silico Analysis 

Sequence data were inspected using Sanger Sequencing and Fragment Analysis Software SeqScape of Applied Biosystems (ThermoFisher Scientific, Waltham, MA, USA). The reference mRNA sequence is NM_002272.4 for *KRT4* and NM_153490.3 for *KRT13* and variants are indicated starting from the first nucleotide of the coding sequence (+1). The reference protein sequence is NP_002263.3 for KRT4 and NP_705694.3 for KRT13. The sequence variants for both genes were searched in gene-specific databases as the Leiden Open Variation database- LOVD (https://databases.lovd.nl/shared/genes/KRT4 and https://databases.lovd.nl/shared/genes/KRT13, accessed 18 May 2022) and the NCBI ClinVar database (https://www.ncbi.nlm.nih.gov/clinvar, accessed 19 May 2022), as well as in general population databases, namely the NCBI Database of Short Genetic Variation–dbSNP (https://www.ncbi.nlm.nih.gov/snp, accessed 25 May 2022) and the Genome Aggregation Database—GnomAD (https://gnomad.broadinstitute.org/, accessed 30 May 2022).

### 2.7. Results Histocytopathological, Immunocytochemistry and Cell Block Results

**Figure 2:**
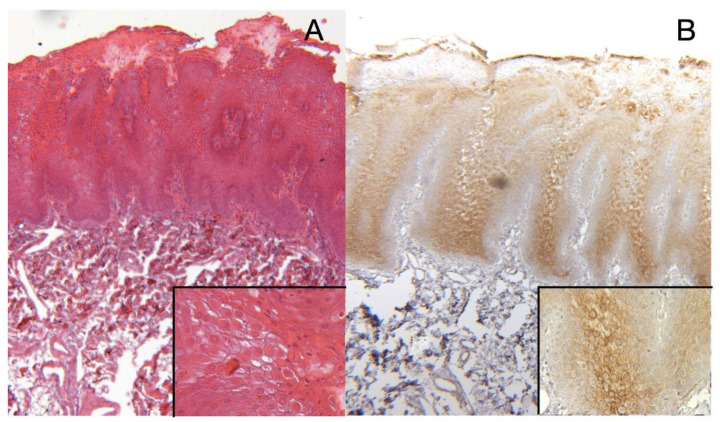
(**A**)—Histological analysis of paraffin-embedded section from oral biopsy shows the presence of edema and acanthosis (H&E 4×); the lower half of the epithelium appears normal. No evidence of dysplasia and no basal cell degeneration. At higher magnification (in the lower box), cells of the prickle cell (spinous) layer displayed marked intracellular edema and nuclear pyknosis (H&E 40×). (**B**)—Overexpression of KRT13 was observed in the same sample (CK13 immunohistochemistry 4× and 40× in the lower box).

Figure 2 displays a paraffin-embedded section from oral biopsy showing the presence of acanthosis and hyper-keratosis of the epithelium: cells of the prickle cell (spinous) layer displayed marked intracellular edema and nuclear pyknosis, while the lower half of the epithelium appeared normal (Figure 2A). There was no evidence of dysplasia and no basal cell degeneration. Figure 2B demonstrates cytokeratin 13 (KRT13) overexpression, confirming the WSN diagnosis for all patients. 

**Figure 3:**
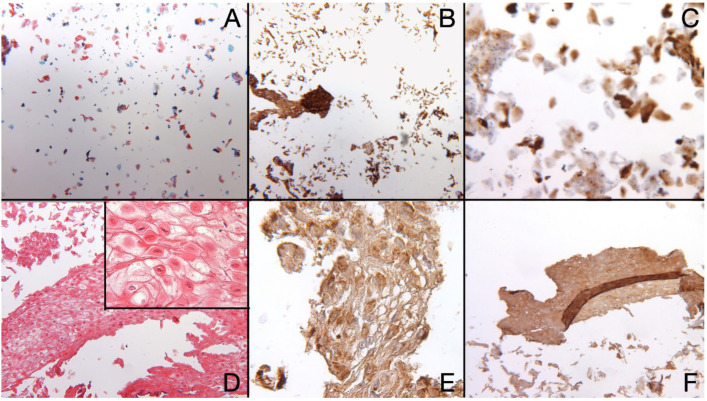
(**A**)—Liquid based cytology from cytobrush with Papanicolaou staining shows scattered cells with no nuclear atypia and no relevant morphological alteration (Cytobrush 4×); (**B**)—Positive immunohistochemical staining for KRT13 (Cytobrush 10×); (**C**)—Positive immunohistochemical staining for KRT13 (Cytobrush 20×); (**D**)—Cell Block shows higher number of cells and aggregates of dyskeratotic keratinocytes from the spinous layer cells and from corneous layer (Cell Block—H&E 10× and 60× in the higher magnification detail); (**E**)—Positive immunohistochemical staining for KRT13 (Cell Block-40×); (**F**)—Positive immunohistochemical staining for KRT4 (Cell Block-20×).

Figure 3 shows the results obtained with oral brushing. Figure 3A corresponds to cytopathology with Papanicolau staining (4×) showing cells with no nuclear atypia and no relevant morphological alterations; overexpression of KRT13 is evident in cytobrush preparations at 10× and 20× (Figure 3B,C). Figure 3D shows the results of Cell Block preparations stained with H&E (Figure 3D) while Figure 3E,F show KRT13 and KRT4 overexpression, respectively. In Figure 3D some cells with cytoplasmic vacuolization and hyperparakeratosis can be noted, while the search for malignant cells was negative. Cell Block preparations show large aggregates of dyskeratotic keratinocytes from the spinous layer cells and from corneous layer (corneocytes) with tenacious aggregation. At higher magnification (inset of Figure 3D), characteristic target features of dyskeratotic keratinocytes can be appreciated. Cell Block preparations are almost indistinguishable from those obtained with oral biopsy and histopathology. 

In conclusion, the brushing method is comparable to conventional histology on oral biopsy sections for reaching a WSN diagnosis. In this specific case, cytobrush samples collected with the Thin Prep Kit have optimal characteristics for analysis, because lesions flake easily. Both histological analysis on oral biopsy and Cell Block preparation on exfoliative cytology led to the same benign diagnosis of WSN, namely hyperkeratotic epithelium with acanthosis and spongiosis. 

### 2.8. Genetic Results

Sequencing analysis in our patients detected 13 rare variants in *KRT4* and *KRT13* genes (Table 3). Most of these variants were listed in gene-specific databases (see Section 2.6 “In Silico Analysis”) and associated to the White Sponge Nevus phenotype. These results confirm the genetic etiology of WSN, completing the diagnostic process and confirming the observations made on the Cell Block specimen. Parents of these patients are healthy but carry approx. 50% of their affected sons’ variants. Most detected *KRT4* and *KRT13* variants are polymorphic in the population, and do not have a direct pathogenetic effect. Only 5 out of 13 variants are missense, while others are either intronic or synonymous; however, each patient has inherited variants from parents in each gene. 

## 3. Discussion

In the last decades, many diagnostic procedures in different fields of medicine took advantage of minimally invasive techniques as cytopathology alone and/or in combination with ancillary techniques (i.e., immune-cytopathology, metabolic assays, genetic sequencing). Nevertheless, in the oral medicine field only recently cytology has been proved as a valid tool for the early diagnosis of oral cancer and biopsy, with an accurate clinical-pathological correlation, remains the gold standard in the diagnosis of oral lesions. In this light, the need of minimally invasive techniques with a high reliability is essential, since many potentially malignant disorders and frankly malignant disorders can affect the oral cavity [7,26,27,28] and non-invasive diagnostic tools are welcome for oral health screenings and follow-up. 

The presentation and thorough diagnostic procedure of these 4 new cases of WSN allowed us to compare non-invasive techniques with the gold standard verified with loupes or microscopy [29,30]. The clinical-pathological correlation in all cases reached the final diagnosis of WSN (white bilateral asymptomatic non-removable plaques in young patients with pathology revealing hyperkeratosis without dysplasia) and the cytopathology results, further confirmed by the results of the genetic analysis, led to the same final diagnosis. 

**Figure 4:**
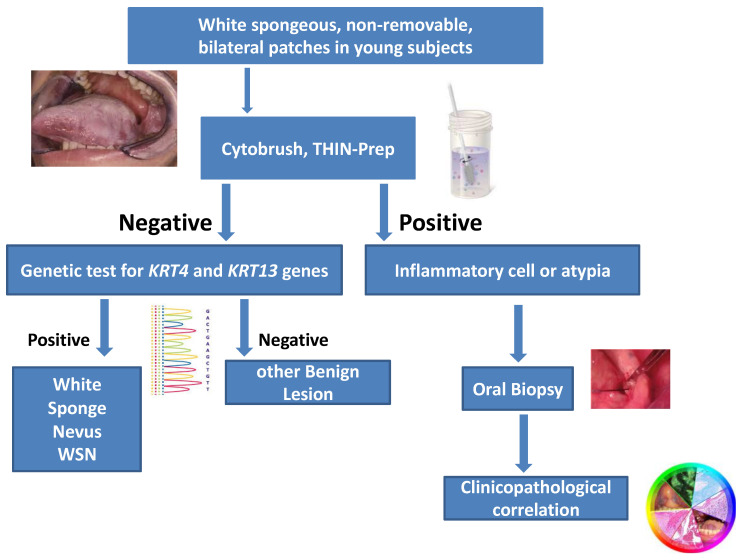
Proposed diagnostic workflow for White Spongeous lesions of the oral cavity.

Even if WSN is considered a benign condition, patients and their parents, in case of young subjects, are worried for the aggressive feature of the oral lesions. Since a final diagnosis of WSN may be reached not only by clinical-pathological correlation performing an oral biopsy, but also comparing the clinical findings with cytopathology results on buccal swab, integrated by genetic analysis, we propose a noninvasive diagnostic algorithm (Figure 4). When observing a child with bilateral not removable asymptomatic oral plaques, performing a buccal swab in order to perform cytopathology and genetic testing of *KRT4* and *KRT13*, may provide a final diagnosis of WSN without performing a biopsy, or at least postponing it until legal age. 

From a clinical point of view, the most obvious advantage of non-invasive techniques is the possibility of performing diagnostic procedure on children or young patients, who may be frightened by the oral biopsy. Furthermore, the non-invasiveness of Thin Prep technique makes it suitable for the follow-up of these patients, leaving the oral biopsy as last resort only if the clinical appearance of the lesions should change. Cytopathology methods are becoming more and more common in the clinical practice thanks to their numerous advantages: they are easy to perform with few tools, samples can be stored up to 30 days in the Thin Prep medium, genetic and other ancillary techniques can be performed on the same samples. Furthermore, recent studies propose Thin Prep technique for screening oral pathologies or tooth anomalies, and as soon as malignant cells are detected, the intervention of the oral surgeon can be immediate [20,31,32].

In adult patients with bilateral white lesion, a different diagnostic algorithm must be followed because malignant disorders are more common and a biopsy is mandatory in the majority of cases. In this context, using the Thin Prep technique to perform a Papanicolau stain could be the first step in order to identify the cell lines (i.e., epithelial, connective, hematological) and oral biopsy could be the second step, in case of malignant cells identification. 

Genetic disorders have specific manifestations and are caused by a derangement of one or more genes with the consequent tissue/organ impairment. Many of these conditions involve the entire body, but in some cases oral signs are the first manifestation of the disorder as in genodermatoses [2,33]. The genetic analysis of our patients was also compatible with the diagnosis of WSN. Furthermore, as shown in Table 3, the DNA variants from both parents eventually combine in our patients, suggesting that the onset of WSN could follow a dose dependent manner i.e., when a certain type and number of variants in the KRT genes reach a given threshold. 

The limit of this research is that is based on the analysis of only four patients, all male. Considering that WSN is a rare disease, their number is still considerable, however, it is desirable to extend the genetic analysis to other WSN cases to confirm that *KRT4* and *KRT13* variants are causative.

## 4. Conclusions

We propose a decision tree for young patients presenting with white oral bilateral asymptomatic non-removable patches, suggesting a provisional diagnosis of WSN. The diagnostic process should start with exfoliative cytopathology and be continued with cell-block preparations and their analysis with traditional staining and additional immunohistochemistry. Genetic analysis should be added in order to characterize putative mutations of *KRT4* or *KRT13*. This non-invasive diagnostic approach is highly reliable and will allow differential diagnosis of WSN without resorting to an invasive diagnostic procedure such as oral biopsy. 

## Figures and Tables

**Table 1:** Differential diagnosis of White Sponge Nevus (WSN) with other genodermatoses with white oral lesions. For each condition (Phenotype), the inheritance patter is indicated (AD: Autosomal Dominant; AR: Autosomal Recessive; XLR: X-linked Recessive; n.a.: not available). When the causative genes are known, they are indicated along their chromosomal location. OMIM stands for Online Mendelian Inheritance in Man, a catalog of human genes with monogenic inheritance (www.omim.org accessed on 14 April 2022).

Inheritance	Phenotype	OMIM	Location	Gene	OMIM
AD	White Sponge Nevus 1; WSN1	#193900	12q13.13	*KRT4*	*123940
AD	White Sponge Nevus 2; WSN2	#615785	17q21.2	*KRT13*	*148065
AD	Hereditary Benign Intraepithelial Dyskeratosis; HBID	%127600	4q35	n.a.	n.a.
	**Pachyonychia congenita, PC**				
AD	Pachyonychia congenita 1/PC1	#167200	17q21.2	*KRT16*	*148067
AD	Pachyonychia congenita 2/PC2	#167210	17q21.2	*KRT17*	*148069
AD	Pachyonychia congenita 3/PC3	#615726	12q13.13	*KRT6A*	*148041
AD	Pachyonychia congenita 4/PC4	#615728	12q13.13	*KRT6B*	*148042
AR	Pachyonychia congenita, autosomal recessive	260130	n.a.	n.a.	n.a.
	**Dyskeratosis congenita, DKC**				
AD	DKC, autosomal dominant 1/DKCA1	#127550	3q26.2	*TERC*	*602322
AD, AR	DKC, autosomal dominant 2/DKCA2 - autosomal recessive 4/DKCB4	#613989	5p15.33	*TERT*	*187270
AD	DKC, autosomal dominant 3/DKCA3 - Revesz syndrome	#613990-#268130	14q12	*TINF2*	*604319
AD, AR	DKC, autosomal dominant 4/DKCA4 - autosomal recessive 5/DKCB5	#615190	20q13.33	*RTEL1*	*608833
AD	DKC, autosomal dominant 6/DKCA6	#616553	16q22.1	*ACD*	*609377
AR	DKC, autosomal recessive 1/DKCB1	#224230	15q14	*NOP10*	*606471
AR	DKC, autosomal recessive 2/DKCB2	#613987	5q35.3	*NHP2*	*606470
AR	DKC, autosomal recessive 3/DKCB3	#613988	17p13.1	*WRAP53*	*612661
AR	DKC, autosomal recessive 6/DKCB6	#616353	16p13.12	*PARN*	*604212
AR	DKC, autosomal recessive 7/DKCB7	#616553	16q22.1	*ACD*	*609377
XLR	DKC, X-linked	#305000	Xq28	*DKC1*	*300126

**Table 2:** List of PCR primer pairs and annealing temperatures used in the present study (F, Forward primer; R, reverse primer) and size of amplicon.

Primers for *KRT4* Gene	Primer (5′ to 3′)	Annealing Temp. (C°)	Amplicon Size (bp)
CK4F1	TGATAGCTCCCAGCTCGCT	55°	603
CK4R1	CCAGGGAAGTTCAGTGGTCT		
CK4F2	TGCCCTGGAGATGCAACATA	55°	464
CK4R2	CCTTCAGAGCCTGAGATTCT		
CK4F3	CCTGGCCTAAACGGGTACTT	55°	332
CK4R3	CCCATGACTTCAGCCAAAGA		
CK4F4	TTCCATGTCTTCAGGTGGCT	55°	402
CK4R4	GATGAGAACCCACTGCCCTA		
CK4F5	TCAGTGAAGGCTTTCCTGG	55°	668 (5F/6R)
CK4R6	TCTGGAGATGTCTGCCTGAG		
CK4F7	TCAGGGAAAGTGGGCAGAA	55°	421
CK47R	GGCATAAATAAGCTCATGGC		
CK4F8	GGTGCATTACGAACCAGGA	55°	650 (8F/9R)
CK4R9	TCCCTGTCCCAGCACAGAA		
**Primers for *KRT13* Gene**	**Primer (5′ to 3′)**	**Annealing** **Temp. (C°)**	**Amplicon Size** **(bp)**
CK13F1	GGGAAGGGAGGAGAGAAGAT	55°	715
CK13R1	CACACCTAGTCCCCCACAA		
CK13F2	TAGCGTATTTGATGTGTTGCC	55°	274
CK13R2	CCAGTGTCATTGGTCAGATG		
CK13F3	ACACTGGAGCATCCCAGGA	55°	668 (3F/4R)
CK13R4	TATGGGATGGGCTATGTGGG		
CK13F5	CTTCCCCACCACCTTCTTC	55°	567 (5F/6R)
CK13R6	TGACATGAGGGGGTGGATC		
CK13F7	GAAATGATAAGCCGAGGCAC	55°	276
CK13R7	CAGTGAGCGAATGACCACTT		
CK13F8	AATGAGGAGTTTGTGAGCCC	55°	301
CK13R8	ACTGAGCCTTGGGTCCAGC		

**Table 3:** List of White Sponge Nevus patients with their respective genetic variants, associated SNP number (rs), parental origin (green: maternal allele; blue: paternal allele) and variant frequencies based on the gnomAD database (gnomad.broadinstitute.org). n.a.: not available.

Patient	Gene	Genetic Variant Effect—(Varsome Classification)	dbSNP (ClinVar)	Parental Origin	Variant Frequency (%)
**P-1**	***KRT4***	c.244_245insTTGGTGGCTTTGGTGCCGGCG GCTTCGGAGCTGGTTTCGGCA p.Gly81_Thr82insIleGlyGlyPheGlyAlaGlyGly PheGlyAlaGlyPheGly in frame insertion—(Uncertain Significance)	rs781060860 (n.a.)	Maternal	2/47694 alleles
		c.678-14G > A intronic variant–(Benign)	rs2307027 (309687)	Maternal	234682/ 280914 alleles (84%)
		c.1345A > G intronic variant–(Benign)	rs931479 (309671)	Paternal	236494/ 282588 alleles (84%)
	***KRT13***	c.114C > T (p.Ser38 =) synonymous variant—(Benign)	rs8182306 (1300084)	Maternal	260852/ 282488 alleles (92%)
		c.735 + 10A > G intronic variant—(Benign)	rs7211835 (323093)	Paternal	257738/ 282656 alleles (91%)
		c.897 + 6C > T intronic variant—(Benign)	rs4796698 (323084)	Paternal	255407/ 282646 alleles (90%)
**P-2**	***KRT4***	c.244_245insTTGGTGGCTTTGGTGCCGGCG GCTTCGGAGCTGGTTTCGGCA p.Gly81_Thr82insIleGlyGlyPheGlyAlaGlyGly PheGlyAlaGlyPheGly in frame insertion—(Uncertain Significance)	rs781060860 (n.a.)	Paternal	2/47694 alleles
		c.678-14G > A intronic variant—(Benign)	rs2307027 (309687)	Maternal	234682/ 280914 alleles (84%)
		c.1020G > C (p.Ser340=) synonymous variant—(Benign)	rs7956809 (309677)	Paternal	27040/ 281468 alleles (10%)
	***KRT13***	c.114C > T (p.Ser38=) synonymous variant—(Benign)	rs8182306 (1300084)	Maternal	260852/ 282488 alleles (92%)
		c.340C > T (p.Arg114Cys) missense variant—(Likely pathogenic)	rs545085703 (n.a.)	Maternal	1/251496 alleles
		c.735 + 10A > G intronic variant—(Benign)	rs7211835 (323093)	Paternal	257738/ 282656 alleles (91%)
		c.897 + 6C > T intronic variant—(Benign)	rs4796698 (323084)	Maternal	255407/ 282646 alleles (90%)
		c.1335T > C (p.Ser445=) synonymous variant—(Likely benign)	rs772942425 (n.a.)	Paternal	2/192196 alleles
**P-3**	***KRT4***	c.244_245insTTGGTGGCTTTGGTGCCGGCG GCTTCGGAGCTGGTTTCGGCA p.Gly81_Thr82insIleGlyGlyPheGlyAlaGlyGly PheGlyAlaGlyPheGly in frame insertion (Uncertain Significance)	rs781060860 (n.a.)	Maternal	2/47694 alleles
		c.678-14G > A intronic variant - (Benign)	rs2307027 (309687)	Paternal	234682/ 280914 alleles (84%)
	***KRT13***	c.114C > T (p.Ser38 =) synonymous variant - (Benign)	rs8182306 (1300084)	Paternal	260852/ 282488 alleles (92%)
		c.437C > G (p.Ala146Gly) missense variant—(Benign)	rs760134 (323101)	Paternal	15256/ 282858 alleles (5.4%)
		c.735 + 10A > G intronic variant—(Benign)	rs7211835 (323093)	Maternal	257738/ 282656 alleles (91%)
		c.897 + 6C > T intronic variant—(Benign)	rs4796698 (323084)	Paternal	255407/ 282646 alleles (90%)
**P-4**	***KRT4***	c.215C > T (p.Ala72Val) missense variant—(Benign)	rs2638525 (309709)	Paternal	49870/ 229154 alleles (21.8%)
		c.467A > G (p.Gln156Arg) missense variant—(Benign)	rs7959052 (309695)	Maternal	53607/ 278106 alleles (19.3%)
	***KRT13***	c.114C > T (p.Ser38 =) synonymous variant—(Benign)	rs8182306 (1300084)	Paternal	260852/ 282488 alleles (92%)
		c.560C > T (p.Ala187Val) missense variant—(Benign)	rs9891361 (323096)	Maternal	239787/ 282512 alleles (85%)
		c.735 + 10A > G intronic variant—(Benign)	rs7211835 (323093)	Paternal	257738/ 282656 alleles (91%)
		c.897 + 6C > T intronic variant—(Benign)	rs4796698 (323084)	Paternal	255407/ 282646 alleles (90%)

## Data Availability

The data that support the findings of this study are available on request from the corresponding author, [P.C.].
